# 2,3,4,6-Tetra-*O*-acetyl-β-d-galacto­pyranosyl 2,3,4,6-tetra-*O*-acetyl-β-d-glucopyranosyl disulfide tetra­hydro­furan solvate

**DOI:** 10.1107/S1600536808039494

**Published:** 2008-11-29

**Authors:** Iván Brito, Lászlo Szilágyi, Matías López-Rodríguez

**Affiliations:** a Departamento de Química, Facultad de Ciencias Básicas, Universidad de Antofagasta, Casilla 170, Antofagasta, Chile; b Department of Organic Chemistry, University of Debrecen, H-4010 Debrecen Pf. 20, Hungary; c Instituto de Bio-Orgánica ’Antonio González’, Universidad de La Laguna, Astrofísico Francisco Sánchez N°2, La Laguna, Tenerife, Spain

## Abstract

The asymmetric unit of title compound, C_28_H_38_O_18_S_2_·C_4_H_8_O, comprises one disulfide-bridged sugar molecule and one solvent molecule. No significant differences in structural parameters are found between the present structure and the previously determined unsolvated form [Brito, López-Rodríguez, Bényei & Szilagyi (2006 ▶). *Carbohydr. Res.* **341**, 2967–2972]. The compounds are characterized by a compact structure with spatial proximity of the two pyranosyl rings. One of the carbonyl atoms is disordered over two sites [site occupancy = 0.69 (7) for major component] and the displacement parameters for the THF species are unsually large.

## Related literature

For analysis of conformation, see: Cremer & Pople (1975 ▶). For the synthesis, see: Szilágyi *et al.* (2001 ▶). For background to disulfide linkage and diglycosyl disulfides, see: André *et al.* (2006 ▶); Chakka *et al.* (2005 ▶); Pérez *et al.* (1978 ▶); Szilágyi & Varela (2006 ▶). For the structure of the unsolvated form, see: Brito *et al.* (2006 ▶).
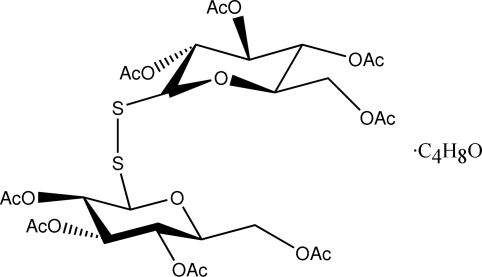


## Experimental

### 

#### Crystal data

C_28_H_38_O_18_S_2_·C_4_H_8_O
*M* *_r_* = 798.81
Monoclinic, 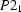

*a* = 14.6499 (14) Å
*b* = 10.0096 (10) Å
*c* = 15.4029 (15) Å
β = 113.573 (2)°
*V* = 2070.2 (4) Å^3^
*Z* = 2
Mo *K*α radiation
μ = 0.20 mm^−1^
*T* = 298 (2) K
0.40 × 0.30 × 0.20 mm

#### Data collection

Nonius KappaCCD area-detector diffractometer
Absorption correction: multi-scan (*SORTAV*; Blessing, 1995 ▶) *T*_min_ = 0.920, *T*_max_ = 0.960
13257 measured reflections
7410 independent reflections
4763 reflections with *I* > 2σ(*I*)
*R*_int_ = 0.046

#### Refinement

*R*[*F* ^2^ > 2σ(*F* ^2^)] = 0.054
*wR*(*F* ^2^) = 0.151
*S* = 0.85
7410 reflections
497 parameters
45 restraints
H-atom parameters constrained
Δρ_max_ = 0.34 e Å^−3^
Δρ_min_ = −0.19 e Å^−3^
Absolute structure: Flack, (1983 ▶), 3438 Friedel pairs
Flack parameter: 0.02 (9)

### 

Data collection: *COLLECT* (Nonius, 2000 ▶); cell refinement: *DENZO-SMN* (Otwinowski & Minor, 1997 ▶); data reduction: *DENZO-SMN*; program(s) used to solve structure: *SIR97* (Altomare *et al.*, 1999 ▶); program(s) used to refine structure: *SHELXL97* (Sheldrick, 2008 ▶); molecular graphics: *ORTEP-3 for Windows* (Farrugia, 1997 ▶) and *PLATON* (Spek, 2003 ▶); software used to prepare material for publication: *WinGX* (Farrugia, 1999 ▶).

## Supplementary Material

Crystal structure: contains datablocks global, I. DOI: 10.1107/S1600536808039494/tk2332sup1.cif

Structure factors: contains datablocks I. DOI: 10.1107/S1600536808039494/tk2332Isup2.hkl

Additional supplementary materials:  crystallographic information; 3D view; checkCIF report

## Figures and Tables

**Table 1:** Selected torsion angle (°)

C1—S1—S2—C21	−80.25 (19)

**Fig. 1.**
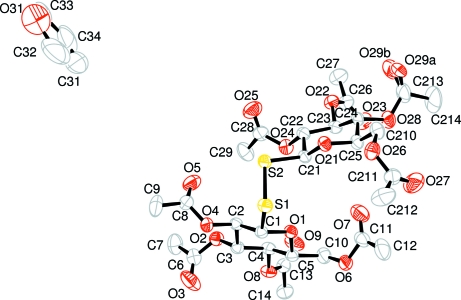
The molecular structure of (I), with the atomic numbering scheme. Displacement ellipsoids are drawn at the 30% probability level. All H atoms have been omitted for clarity.

## supplementary crystallographic information

### Comment

The disulfide linkage which plays an essential role in stabilizing the tertiary structure of proteins, was not known in carbohydrate chemistry until recently when it was introduced as a new interglycosidic connecting element (Szilágyi & Varela, 2006). Diglycosyl disulfides were proposed as novel carbohydrate scaffolds with potential biological activity (Szilágyi & Varela, 2006, Chakka *et al.*, 2005). This has recently been demonstrated in binding studies with galectins and in assays with tumor cells (André *et al.*, 2006). We report herein the crystal and molecular structure of the title compound, (I), Fig. 1. The averaged bond lengths [C—O 1.429 (6), O—C 1.341 (8), C=O 1.181 (8), C—C 1.478 (10) Å] and other parameters, Table 1, compare well with those for the previously determined unsolvated form (Brito *et al.*, 2006). The pyranosyl rings adopt chair conformations with Cremer & Pople (1975) puckering parameters: Q_T _= 0.604 (6) (Glc) and 0.566 (5) Å (Gal), θ = 2.4 (5) (Glc) and 9.6 (6)°(Gal), φ = 15 (11) (Glc) and 9(4)° (Gal); these parameters are similar to those for the unsolvated form. The conformations of the acetyl groups are in agreement with the observation that in acetylated pyranoses, the carbonyl C=O bonds tend to align so that they nearly eclipse the axial-H atoms atoms on the common ring C atoms.

### Experimental

Compound (I) was synthesized as described by Szilágyi *et al.* (2001).

### Refinement

The H atoms were geometrically placed (C—H = 0.96–0.98 Å) and refined as riding with *U*_iso_(H) = 1.2*U*_eq_(C) or 1.5*U*_eq_(methyl-C). The O29 atom was refined over two sites using a disorder model, with occupancies of 0.69 (7) and 0.31 (7). The THF molecule is disordered and was modelled with restraints for distances and anisotropic displacement parameters using a 'rigid-bond' restraint to *U^ij^*, implemented as the DELU instruction in *SHELXL97* (Sheldrick, 2008).

### Crystal data

**Table:**

C_28_H_38_O_18_S_2_·C_4_H_8_O	*F*_000_ = 844
*M**_r_* = 798.81	*D*_x_ = 1.281 Mg m^−^^3^
Monoclinic, *P*2_1_	Mo *K*α radiation λ = 0.71073 Å
Hall symbol: P 2yb	Cell parameters from 4312 reflections
*a* = 14.6499 (14) Å	θ = 2.4–25.0º
*b* = 10.0096 (10) Å	µ = 0.20 mm^−^^1^
*c* = 15.4029 (15) Å	*T* = 298 (2) K
β = 113.573 (2)º	Prism, yellow
*V* = 2070.2 (4) Å^3^	0.40 × 0.30 × 0.20 mm
*Z* = 2	

### Data collection

**Table:**

Nonius KappaCCD area-detector diffractometer	7410 independent reflections
Radiation source: fine-focus sealed tube	4763 reflections with *I* > 2σ(*I*)
Monochromator: graphite	*R*_int_ = 0.046
*T* = 298(2) K	θ_max_ = 25.2º
φ scans, and ω scans with κ offsets	θ_min_ = 2.5º
Absorption correction: multi-scan(SORTAV; Blessing, 1995)	*h* = −17→16
*T*_min_ = 0.920, *T*_max_ = 0.960	*k* = −12→11
13257 measured reflections	*l* = −18→18

### Refinement

**Table:**

Refinement on *F*^2^	Hydrogen site location: inferred from neighbouring sites
Least-squares matrix: full	H-atom parameters constrained
*R*[*F*^2^ > 2σ(*F*^2^)] = 0.054	*w* = 1/[σ^2^(*F*_o_^2^) + (0.0875*P*)^2^] where *P* = (*F*_o_^2^ + 2*F*_c_^2^)/3
*wR*(*F*^2^) = 0.151	(Δ/σ)_max_ = 0.042
*S* = 0.85	Δρ_max_ = 0.34 e Å^−^^3^
7410 reflections	Δρ_min_ = −0.19 e Å^−^^3^
497 parameters	Extinction correction: none
45 restraints	Absolute structure: Flack, (1983), 3438 Friedel pairs
Primary atom site location: structure-invariant direct methods	Flack parameter: 0.02 (9)
Secondary atom site location: difference Fourier map	

### Special details

**Table:**

Geometry. All e.s.d.'s (except the e.s.d. in the dihedral angle between two l.s. planes) are estimated using the full covariance matrix. The cell e.s.d.'s are taken into account individually in the estimation of e.s.d.'s in distances, angles and torsion angles; correlations between e.s.d.'s in cell parameters are only used when they are defined by crystal symmetry. An approximate (isotropic) treatment of cell e.s.d.'s is used for estimating e.s.d.'s involving l.s. planes.
Refinement. Refinement of *F*^2^ against ALL reflections. The weighted *R*-factor *wR* and goodness of fit *S* are based on *F*^2^, conventional *R*-factors *R* are based on *F*, with *F* set to zero for negative *F*^2^. The threshold expression of *F*^2^ > σ(*F*^2^) is used only for calculating *R*-factors(gt) *etc*. and is not relevant to the choice of reflections for refinement. *R*-factors based on *F*^2^ are statistically about twice as large as those based on *F*, and *R*- factors based on ALL data will be even larger.

### Fractional atomic coordinates and isotropic or equivalent isotropic displacement parameters (Å^2^)

**Table:**

	*x*	*y*	*z*	*U*_iso_*/*U*_eq_	Occ. (<1)
S1	0.94859 (8)	0.98543 (12)	0.93468 (8)	0.0609 (3)	
O1	0.8235 (2)	1.1214 (3)	0.98368 (19)	0.0587 (7)	
O2	0.5745 (2)	1.1063 (3)	0.7358 (2)	0.0692 (8)	
O3	0.5648 (4)	1.2681 (6)	0.6357 (3)	0.146 (2)	
O4	0.7645 (2)	1.0285 (3)	0.7398 (2)	0.0644 (8)	
O5	0.6752 (4)	0.8414 (4)	0.7054 (3)	0.1135 (14)	
O6	0.8497 (3)	1.2956 (4)	1.1307 (2)	0.0822 (9)	
O7	0.8713 (4)	1.0964 (5)	1.2001 (3)	0.1132 (14)	
O8	0.5850 (2)	1.2941 (3)	0.8764 (2)	0.0684 (8)	
O9	0.5145 (3)	1.1965 (4)	0.9648 (3)	0.1042 (13)	
C1	0.8424 (3)	1.0964 (4)	0.9008 (3)	0.0546 (10)	
H1	0.8612	1.1814	0.8809	0.066*	
C2	0.7471 (3)	1.0494 (4)	0.8225 (3)	0.0548 (10)	
H2	0.7253	0.9658	0.8412	0.066*	
C3	0.6682 (3)	1.1554 (4)	0.8047 (3)	0.0580 (11)	
H3	0.6874	1.2365	0.7806	0.07*	
C4	0.6549 (3)	1.1872 (5)	0.8953 (3)	0.0624 (11)	
H4	0.6301	1.1083	0.9166	0.075*	
C5	0.7552 (3)	1.2286 (4)	0.9704 (3)	0.0601 (11)	
H5	0.7793	1.3079	0.9487	0.072*	
C6	0.5307 (4)	1.1702 (7)	0.6531 (4)	0.0865 (16)	
C7	0.4395 (5)	1.1036 (8)	0.5895 (4)	0.116 (2)	
H7A	0.3886	1.1144	0.6135	0.174*	
H7B	0.4524	1.0102	0.5859	0.174*	
H7C	0.4175	1.1426	0.5275	0.174*	
C8	0.7260 (4)	0.9197 (6)	0.6875 (4)	0.0773 (14)	
C9	0.7533 (5)	0.9114 (8)	0.6047 (4)	0.116 (2)	
H9A	0.7772	0.8231	0.6009	0.175*	
H9B	0.8046	0.9754	0.6117	0.175*	
H9C	0.6959	0.9302	0.5478	0.175*	
C10	0.7520 (4)	1.2569 (6)	1.0644 (3)	0.0802 (15)	
H10A	0.7049	1.3282	1.058	0.096*	
H10B	0.7302	1.1779	1.0873	0.096*	
C11	0.9038 (5)	1.2052 (7)	1.1941 (4)	0.0896 (17)	
C12	1.0060 (5)	1.2548 (8)	1.2510 (4)	0.122 (2)	
H12A	1.0442	1.1855	1.2929	0.183*	
H12B	1.0025	1.3309	1.2875	0.183*	
H12C	1.0374	1.2801	1.2094	0.183*	
C13	0.5199 (3)	1.2900 (6)	0.9193 (3)	0.0704 (12)	
C14	0.4608 (4)	1.4138 (5)	0.9033 (4)	0.0890 (16)	
H14A	0.4028	1.3978	0.9161	0.133*	
H14B	0.4408	1.4417	0.8386	0.133*	
H14C	0.5005	1.4826	0.9447	0.133*	
S2	0.89370 (8)	0.80485 (11)	0.95029 (7)	0.0611 (3)	
O21	0.98609 (19)	0.7760 (3)	1.13641 (18)	0.0584 (7)	
O22	0.7552 (2)	0.5961 (3)	1.1697 (2)	0.0684 (8)	
O23	0.6851 (3)	0.7007 (4)	1.2553 (3)	0.0950 (11)	
O24	0.7182 (2)	0.7573 (3)	1.0062 (2)	0.0678 (8)	
O25	0.6962 (4)	0.5714 (5)	0.9230 (3)	0.1235 (16)	
O26	1.1652 (2)	0.8389 (4)	1.2729 (2)	0.0751 (9)	
O27	1.1690 (4)	0.9965 (5)	1.3749 (4)	0.1336 (17)	
O28	0.9183 (2)	0.7190 (4)	1.3348 (2)	0.0803 (9)	
O29A	0.988 (5)	0.519 (4)	1.3922 (18)	0.103 (15)	0.31 (7)
O29B	0.921 (4)	0.5063 (16)	1.3697 (18)	0.155 (9)	0.69 (7)
C21	0.8909 (3)	0.8131 (4)	1.0674 (3)	0.0531 (9)	
H21	0.8739	0.9037	1.0799	0.064*	
C22	0.8144 (3)	0.7149 (4)	1.0712 (3)	0.0576 (10)	
H22	0.8288	0.626	1.0531	0.069*	
C23	0.8154 (3)	0.7094 (5)	1.1694 (3)	0.0601 (11)	
H23	0.7853	0.791	1.1815	0.072*	
C24	0.9198 (3)	0.6941 (4)	1.2442 (3)	0.0593 (11)	
H24	0.943	0.6028	1.2424	0.071*	
C25	0.9914 (3)	0.7911 (5)	1.2309 (3)	0.0593 (10)	
H25	0.974	0.8827	1.2407	0.071*	
C26	0.6918 (4)	0.6043 (5)	1.2123 (4)	0.0740 (13)	
C27	0.6347 (5)	0.4806 (6)	1.2017 (5)	0.1028 (18)	
H27A	0.584	0.494	1.2256	0.154*	
H27B	0.6785	0.4103	1.2367	0.154*	
H27C	0.6042	0.4566	1.136	0.154*	
C28	0.6681 (4)	0.6785 (6)	0.9334 (4)	0.0798 (14)	
C29	0.5757 (4)	0.7449 (7)	0.8683 (4)	0.105 (2)	
H29A	0.5918	0.8306	0.8502	0.157*	
H29B	0.5313	0.7564	0.8997	0.157*	
H29C	0.5441	0.6907	0.8129	0.157*	
C210	1.0967 (3)	0.7613 (5)	1.2966 (3)	0.0708 (13)	
H21A	1.1056	0.7808	1.3612	0.085*	
H21B	1.1102	0.6671	1.2928	0.085*	
C211	1.1968 (4)	0.9552 (5)	1.3177 (4)	0.0830 (15)	
C212	1.2625 (6)	1.0235 (7)	1.2826 (6)	0.128 (2)	
H21C	1.2737	1.1132	1.3067	0.191*	
H21D	1.2324	1.0257	1.2146	0.191*	
H21E	1.3249	0.9769	1.3032	0.191*	
C213	0.9328 (8)	0.6160 (10)	1.3954 (5)	0.115 (3)	
C214	0.9212 (7)	0.6644 (11)	1.4834 (5)	0.174 (4)	
H21F	0.9701	0.6219	1.5381	0.261*	
H21G	0.8557	0.6426	1.4792	0.261*	
H21H	0.9304	0.7595	1.4888	0.261*	
O31	0.7418 (11)	0.0601 (14)	0.3640 (9)	0.306 (5)	
C31	0.6827 (15)	0.2535 (13)	0.4405 (8)	0.301 (9)	
H31A	0.6559	0.3386	0.4113	0.361*	
H31B	0.71	0.265	0.5087	0.361*	
C32	0.7705 (12)	0.2047 (18)	0.4071 (10)	0.291 (7)	
H32A	0.7751	0.2654	0.3599	0.349*	
H32B	0.8343	0.203	0.4608	0.349*	
C33	0.6337 (11)	0.0251 (15)	0.3603 (6)	0.235 (5)	
H33A	0.5878	0.0138	0.2947	0.282*	
H33B	0.637	−0.059	0.3927	0.282*	
C34	0.5917 (12)	0.1382 (19)	0.4080 (8)	0.281 (6)	
H34A	0.5818	0.1036	0.4624	0.337*	
H34B	0.5293	0.1746	0.363	0.337*	

### Atomic displacement parameters (Å^2^)

**Table:**

	*U*^11^	*U*^22^	*U*^33^	*U*^12^	*U*^13^	*U*^23^
S1	0.0625 (6)	0.0635 (6)	0.0677 (7)	0.0038 (6)	0.0377 (5)	0.0038 (6)
O1	0.0704 (18)	0.0570 (17)	0.0581 (17)	0.0048 (13)	0.0358 (15)	0.0102 (15)
O2	0.0641 (19)	0.0688 (19)	0.071 (2)	−0.0002 (16)	0.0228 (16)	−0.0020 (16)
O3	0.132 (4)	0.165 (5)	0.098 (3)	0.056 (3)	0.001 (3)	−0.036 (4)
O4	0.081 (2)	0.0657 (19)	0.0530 (17)	−0.0031 (15)	0.0335 (15)	−0.0036 (16)
O5	0.157 (4)	0.073 (3)	0.121 (3)	−0.025 (2)	0.066 (3)	−0.029 (3)
O6	0.110 (3)	0.070 (2)	0.0621 (19)	0.0011 (18)	0.0296 (19)	0.023 (2)
O7	0.144 (4)	0.103 (3)	0.106 (3)	0.029 (3)	0.064 (3)	0.023 (3)
O8	0.0714 (19)	0.0543 (17)	0.089 (2)	0.0138 (16)	0.0425 (17)	0.0106 (16)
O9	0.117 (3)	0.098 (3)	0.135 (3)	0.041 (3)	0.090 (3)	0.031 (2)
C1	0.063 (3)	0.051 (2)	0.059 (2)	0.004 (2)	0.033 (2)	0.000 (2)
C2	0.061 (3)	0.053 (2)	0.055 (2)	0.0059 (19)	0.028 (2)	−0.002 (2)
C3	0.056 (3)	0.054 (2)	0.062 (3)	0.002 (2)	0.021 (2)	−0.002 (2)
C4	0.063 (3)	0.058 (3)	0.073 (3)	0.011 (2)	0.034 (2)	0.007 (2)
C5	0.075 (3)	0.048 (2)	0.066 (3)	0.004 (2)	0.037 (2)	0.008 (2)
C6	0.088 (4)	0.098 (4)	0.071 (4)	0.003 (3)	0.030 (3)	0.001 (3)
C7	0.092 (4)	0.161 (6)	0.077 (4)	−0.009 (4)	0.016 (3)	−0.015 (4)
C8	0.090 (4)	0.069 (3)	0.067 (3)	−0.005 (3)	0.025 (3)	0.003 (3)
C9	0.137 (6)	0.140 (6)	0.069 (4)	−0.033 (4)	0.039 (4)	0.000 (5)
C10	0.087 (4)	0.087 (4)	0.071 (3)	0.001 (3)	0.036 (3)	0.020 (3)
C11	0.125 (5)	0.082 (4)	0.076 (4)	0.014 (3)	0.054 (4)	0.030 (4)
C12	0.116 (5)	0.149 (7)	0.079 (4)	0.012 (4)	0.017 (4)	0.024 (5)
C13	0.069 (3)	0.075 (3)	0.074 (3)	0.000 (3)	0.036 (2)	0.006 (3)
C14	0.084 (4)	0.081 (4)	0.108 (4)	−0.003 (3)	0.046 (3)	0.013 (3)
S2	0.0789 (7)	0.0549 (6)	0.0570 (6)	0.0025 (5)	0.0350 (5)	0.0060 (6)
O21	0.0597 (17)	0.0695 (19)	0.0547 (16)	0.0044 (13)	0.0320 (14)	0.0082 (14)
O22	0.080 (2)	0.0643 (18)	0.080 (2)	−0.0035 (16)	0.0514 (18)	−0.0061 (17)
O23	0.105 (3)	0.095 (3)	0.118 (3)	−0.019 (2)	0.079 (2)	−0.014 (2)
O24	0.0570 (18)	0.079 (2)	0.0675 (18)	−0.0007 (16)	0.0246 (16)	0.0021 (16)
O25	0.129 (4)	0.086 (3)	0.124 (4)	−0.026 (3)	0.017 (3)	−0.016 (3)
O26	0.070 (2)	0.091 (3)	0.0701 (19)	−0.0123 (18)	0.0342 (16)	−0.0047 (18)
O27	0.186 (5)	0.107 (3)	0.133 (4)	−0.030 (3)	0.090 (4)	0.005 (4)
O28	0.095 (2)	0.097 (2)	0.0640 (19)	0.0000 (19)	0.0475 (18)	0.002 (2)
O29A	0.14 (3)	0.077 (17)	0.066 (10)	0.025 (8)	0.016 (13)	0.010 (13)
O29B	0.23 (3)	0.128 (9)	0.093 (8)	0.030 (6)	0.052 (14)	−0.045 (10)
C21	0.056 (2)	0.054 (2)	0.052 (2)	0.005 (2)	0.0246 (19)	0.012 (2)
C22	0.064 (3)	0.055 (2)	0.060 (2)	0.005 (2)	0.031 (2)	0.009 (2)
C23	0.067 (3)	0.058 (3)	0.068 (3)	0.005 (2)	0.041 (2)	0.010 (2)
C24	0.078 (3)	0.061 (3)	0.051 (2)	0.006 (2)	0.038 (2)	0.011 (2)
C25	0.062 (3)	0.069 (3)	0.054 (2)	0.002 (2)	0.031 (2)	0.006 (2)
C26	0.081 (3)	0.070 (3)	0.092 (3)	−0.006 (3)	0.055 (3)	−0.001 (3)
C27	0.124 (5)	0.090 (4)	0.130 (5)	−0.011 (4)	0.088 (4)	−0.024 (4)
C28	0.074 (4)	0.075 (4)	0.087 (4)	0.000 (3)	0.028 (3)	−0.024 (3)
C29	0.073 (3)	0.137 (5)	0.086 (4)	0.028 (4)	0.013 (3)	−0.014 (4)
C210	0.067 (3)	0.091 (4)	0.062 (3)	0.005 (2)	0.034 (2)	0.007 (3)
C211	0.094 (4)	0.072 (4)	0.082 (4)	−0.001 (3)	0.035 (3)	0.016 (3)
C212	0.145 (6)	0.086 (4)	0.176 (7)	−0.019 (4)	0.089 (6)	−0.021 (4)
C213	0.156 (8)	0.133 (7)	0.066 (4)	0.007 (5)	0.055 (4)	−0.037 (7)
C214	0.240 (10)	0.230 (11)	0.092 (5)	−0.013 (6)	0.109 (6)	−0.051 (8)
O31	0.397 (14)	0.285 (12)	0.252 (10)	0.024 (9)	0.148 (11)	0.016 (11)
C31	0.48 (2)	0.171 (9)	0.103 (7)	0.016 (6)	−0.034 (11)	0.121 (11)
C32	0.372 (14)	0.227 (12)	0.150 (11)	0.082 (9)	−0.024 (10)	0.003 (12)
C33	0.352 (13)	0.214 (10)	0.069 (5)	0.032 (5)	0.011 (7)	0.003 (10)
C34	0.376 (13)	0.332 (17)	0.094 (7)	0.074 (8)	0.050 (9)	0.126 (12)

### Geometric parameters (Å, °)

**Table:**

S1—C1	1.810 (4)	O24—C22	1.429 (5)
S1—S2	2.0313 (16)	O25—C28	1.182 (7)
O1—C5	1.425 (5)	O26—C211	1.339 (6)
O1—C1	1.432 (4)	O26—C210	1.427 (5)
O2—C6	1.338 (7)	O27—C211	1.183 (6)
O2—C3	1.445 (5)	O28—C213	1.349 (8)
O3—C6	1.178 (7)	O28—C24	1.427 (5)
O4—C8	1.337 (6)	O29A—C213	1.27 (3)
O4—C2	1.410 (5)	O29B—C213	1.156 (17)
O5—C8	1.187 (6)	C21—C22	1.509 (6)
O6—C11	1.335 (6)	C21—H21	0.98
O6—C10	1.440 (6)	C22—C23	1.509 (6)
O7—C11	1.207 (7)	C22—H22	0.98
O8—C13	1.361 (5)	C23—C24	1.510 (6)
O8—C4	1.428 (5)	C23—H23	0.98
O9—C13	1.191 (6)	C24—C25	1.503 (6)
C1—C2	1.510 (6)	C24—H24	0.98
C1—H1	0.98	C25—C210	1.497 (6)
C2—C3	1.511 (6)	C25—H25	0.98
C2—H2	0.98	C26—C27	1.466 (7)
C3—C4	1.517 (6)	C27—H27A	0.96
C3—H3	0.98	C27—H27B	0.96
C4—C5	1.520 (6)	C27—H27C	0.96
C4—H4	0.98	C28—C29	1.481 (7)
C5—C10	1.495 (6)	C29—H29A	0.96
C5—H5	0.98	C29—H29B	0.96
C6—C7	1.463 (8)	C29—H29C	0.96
C7—H7A	0.96	C210—H21A	0.97
C7—H7B	0.96	C210—H21B	0.97
C7—H7C	0.96	C211—C212	1.450 (9)
C8—C9	1.484 (8)	C212—H21C	0.96
C9—H9A	0.96	C212—H21D	0.96
C9—H9B	0.96	C212—H21E	0.96
C9—H9C	0.96	C213—C214	1.513 (9)
C10—H10A	0.97	C214—H21F	0.96
C10—H10B	0.97	C214—H21G	0.96
C11—C12	1.486 (9)	C214—H21H	0.96
C12—H12A	0.96	O31—C32	1.578 (16)
C12—H12B	0.96	O31—C33	1.601 (14)
C12—H12C	0.96	C31—C32	1.640 (17)
C13—C14	1.475 (7)	C31—C34	1.68 (2)
C14—H14A	0.96	C31—H31A	0.97
C14—H14B	0.96	C31—H31B	0.97
C14—H14C	0.96	C32—H32A	0.97
S2—C21	1.823 (4)	C32—H32B	0.97
O21—C21	1.424 (4)	C33—C34	1.601 (15)
O21—C25	1.434 (4)	C33—H33A	0.97
O22—C26	1.337 (5)	C33—H33B	0.97
O22—C23	1.437 (5)	C34—H34A	0.97
O23—C26	1.196 (6)	C34—H34B	0.97
O24—C28	1.326 (6)		
			
C1—S1—S2	104.17 (14)	C21—C22—C23	110.7 (4)
C5—O1—C1	112.2 (3)	O24—C22—H22	109.5
C6—O2—C3	119.4 (4)	C21—C22—H22	109.5
C8—O4—C2	118.8 (4)	C23—C22—H22	109.5
C11—O6—C10	117.9 (5)	O22—C23—C22	105.8 (3)
C13—O8—C4	117.6 (3)	O22—C23—C24	110.7 (4)
O1—C1—C2	108.7 (3)	C22—C23—C24	111.6 (3)
O1—C1—S1	107.5 (3)	O22—C23—H23	109.6
C2—C1—S1	116.9 (3)	C22—C23—H23	109.6
O1—C1—H1	107.8	C24—C23—H23	109.6
C2—C1—H1	107.8	O28—C24—C25	107.8 (3)
S1—C1—H1	107.8	O28—C24—C23	108.8 (3)
O4—C2—C1	109.0 (3)	C25—C24—C23	112.4 (3)
O4—C2—C3	110.0 (3)	O28—C24—H24	109.3
C1—C2—C3	108.8 (3)	C25—C24—H24	109.3
O4—C2—H2	109.7	C23—C24—H24	109.3
C1—C2—H2	109.7	O21—C25—C210	106.7 (3)
C3—C2—H2	109.7	O21—C25—C24	108.2 (3)
O2—C3—C2	109.1 (3)	C210—C25—C24	111.5 (4)
O2—C3—C4	108.3 (3)	O21—C25—H25	110.1
C2—C3—C4	110.6 (3)	C210—C25—H25	110.1
O2—C3—H3	109.6	C24—C25—H25	110.1
C2—C3—H3	109.6	O23—C26—O22	123.2 (5)
C4—C3—H3	109.6	O23—C26—C27	125.0 (4)
O8—C4—C3	108.4 (3)	O22—C26—C27	111.8 (4)
O8—C4—C5	110.3 (3)	C26—C27—H27A	109.5
C3—C4—C5	108.4 (3)	C26—C27—H27B	109.5
O8—C4—H4	109.9	H27A—C27—H27B	109.5
C3—C4—H4	109.9	C26—C27—H27C	109.5
C5—C4—H4	109.9	H27A—C27—H27C	109.5
O1—C5—C10	107.2 (3)	H27B—C27—H27C	109.5
O1—C5—C4	108.3 (3)	O25—C28—O24	123.3 (5)
C10—C5—C4	113.1 (4)	O25—C28—C29	126.1 (6)
O1—C5—H5	109.4	O24—C28—C29	110.6 (5)
C10—C5—H5	109.4	C28—C29—H29A	109.5
C4—C5—H5	109.4	C28—C29—H29B	109.5
O3—C6—O2	121.9 (6)	H29A—C29—H29B	109.5
O3—C6—C7	125.7 (6)	C28—C29—H29C	109.5
O2—C6—C7	112.4 (6)	H29A—C29—H29C	109.5
C6—C7—H7A	109.5	H29B—C29—H29C	109.5
C6—C7—H7B	109.5	O26—C210—C25	111.0 (4)
H7A—C7—H7B	109.5	O26—C210—H21A	109.4
C6—C7—H7C	109.5	C25—C210—H21A	109.4
H7A—C7—H7C	109.5	O26—C210—H21B	109.4
H7B—C7—H7C	109.5	C25—C210—H21B	109.4
O5—C8—O4	123.3 (5)	H21A—C210—H21B	108
O5—C8—C9	124.9 (6)	O27—C211—O26	122.4 (6)
O4—C8—C9	111.8 (5)	O27—C211—C212	126.2 (6)
C8—C9—H9A	109.5	O26—C211—C212	111.2 (5)
C8—C9—H9B	109.5	C211—C212—H21C	109.5
H9A—C9—H9B	109.5	C211—C212—H21D	109.5
C8—C9—H9C	109.5	H21C—C212—H21D	109.5
H9A—C9—H9C	109.5	C211—C212—H21E	109.5
H9B—C9—H9C	109.5	H21C—C212—H21E	109.5
O6—C10—C5	109.3 (4)	H21D—C212—H21E	109.5
O6—C10—H10A	109.8	O29B—C213—O29A	43.8 (10)
C5—C10—H10A	109.8	O29B—C213—O28	121.9 (11)
O6—C10—H10B	109.8	O29A—C213—O28	118.7 (18)
C5—C10—H10B	109.8	O29B—C213—C214	123.7 (11)
H10A—C10—H10B	108.3	O29A—C213—C214	125.3 (12)
O7—C11—O6	122.0 (6)	O28—C213—C214	109.4 (8)
O7—C11—C12	126.3 (6)	C213—C214—H21F	109.5
O6—C11—C12	111.6 (6)	C213—C214—H21G	109.5
C11—C12—H12A	109.5	H21F—C214—H21G	109.5
C11—C12—H12B	109.5	C213—C214—H21H	109.5
H12A—C12—H12B	109.5	H21F—C214—H21H	109.5
C11—C12—H12C	109.5	H21G—C214—H21H	109.5
H12A—C12—H12C	109.5	C32—O31—C33	108.0 (14)
H12B—C12—H12C	109.5	C32—C31—C34	109.3 (9)
O9—C13—O8	122.3 (4)	C32—C31—H31A	109.8
O9—C13—C14	125.9 (4)	C34—C31—H31A	109.8
O8—C13—C14	111.8 (5)	C32—C31—H31B	109.8
C13—C14—H14A	109.5	C34—C31—H31B	109.8
C13—C14—H14B	109.5	H31A—C31—H31B	108.3
H14A—C14—H14B	109.5	O31—C32—C31	107.2 (12)
C13—C14—H14C	109.5	O31—C32—H32A	110.3
H14A—C14—H14C	109.5	C31—C32—H32A	110.3
H14B—C14—H14C	109.5	O31—C32—H32B	110.3
C21—S2—S1	104.10 (15)	C31—C32—H32B	110.3
C21—O21—C25	111.7 (3)	H32A—C32—H32B	108.5
C26—O22—C23	120.0 (4)	C34—C33—O31	112.7 (12)
C28—O24—C22	118.2 (4)	C34—C33—H33A	109.1
C211—O26—C210	118.4 (4)	O31—C33—H33A	109
C213—O28—C24	118.9 (5)	C34—C33—H33B	109.1
O21—C21—C22	108.8 (3)	O31—C33—H33B	109
O21—C21—S2	108.9 (2)	H33A—C33—H33B	107.8
C22—C21—S2	108.6 (3)	C33—C34—C31	102.6 (10)
O21—C21—H21	110.2	C33—C34—H34A	111.2
C22—C21—H21	110.2	C31—C34—H34A	111.3
S2—C21—H21	110.2	C33—C34—H34B	111.2
O24—C22—C21	108.6 (3)	C31—C34—H34B	111.3
O24—C22—C23	108.9 (3)	H34A—C34—H34B	109.2
			
C5—O1—C1—C2	−64.8 (4)	S1—S2—C21—C22	156.3 (2)
C5—O1—C1—S1	167.8 (3)	C28—O24—C22—C21	116.5 (4)
S2—S1—C1—O1	74.3 (3)	C28—O24—C22—C23	−122.8 (4)
S2—S1—C1—C2	−48.2 (3)	O21—C21—C22—O24	176.3 (3)
C8—O4—C2—C1	137.4 (4)	S2—C21—C22—O24	−65.2 (3)
C8—O4—C2—C3	−103.4 (4)	O21—C21—C22—C23	56.8 (4)
O1—C1—C2—O4	178.3 (3)	S2—C21—C22—C23	175.2 (3)
S1—C1—C2—O4	−59.9 (4)	C26—O22—C23—C22	−138.6 (4)
O1—C1—C2—C3	58.3 (4)	C26—O22—C23—C24	100.4 (4)
S1—C1—C2—C3	−179.9 (3)	O24—C22—C23—O22	71.7 (4)
C6—O2—C3—C2	−118.1 (4)	C21—C22—C23—O22	−169.0 (3)
C6—O2—C3—C4	121.5 (4)	O24—C22—C23—C24	−167.9 (4)
O4—C2—C3—O2	65.7 (4)	C21—C22—C23—C24	−48.5 (5)
C1—C2—C3—O2	−175.0 (3)	C213—O28—C24—C25	−128.2 (6)
O4—C2—C3—C4	−175.3 (3)	C213—O28—C24—C23	109.8 (6)
C1—C2—C3—C4	−56.0 (4)	O22—C23—C24—O28	−75.1 (4)
C13—O8—C4—C3	142.4 (4)	C22—C23—C24—O28	167.4 (4)
C13—O8—C4—C5	−99.0 (4)	O22—C23—C24—C25	165.7 (3)
O2—C3—C4—O8	−64.5 (4)	C22—C23—C24—C25	48.1 (5)
C2—C3—C4—O8	175.9 (3)	C21—O21—C25—C210	−175.0 (4)
O2—C3—C4—C5	175.6 (3)	C21—O21—C25—C24	64.9 (4)
C2—C3—C4—C5	56.1 (4)	O28—C24—C25—O21	−174.4 (3)
C1—O1—C5—C10	−172.6 (4)	C23—C24—C25—O21	−54.6 (4)
C1—O1—C5—C4	65.0 (4)	O28—C24—C25—C210	68.6 (4)
O8—C4—C5—O1	−177.4 (3)	C23—C24—C25—C210	−171.6 (4)
C3—C4—C5—O1	−58.8 (4)	C23—O22—C26—O23	−3.7 (8)
O8—C4—C5—C10	63.9 (5)	C23—O22—C26—C27	177.8 (4)
C3—C4—C5—C10	−177.6 (4)	C22—O24—C28—O25	5.0 (7)
C3—O2—C6—O3	−2.4 (8)	C22—O24—C28—C29	−173.7 (4)
C3—O2—C6—C7	176.9 (4)	C211—O26—C210—C25	93.8 (5)
C2—O4—C8—O5	2.4 (7)	O21—C25—C210—O26	52.3 (5)
C2—O4—C8—C9	−178.6 (4)	C24—C25—C210—O26	170.2 (4)
C11—O6—C10—C5	−102.8 (5)	C210—O26—C211—O27	−1.1 (8)
O1—C5—C10—O6	60.9 (5)	C210—O26—C211—C212	−177.6 (5)
C4—C5—C10—O6	−179.8 (4)	C24—O28—C213—O29B	−20 (3)
C10—O6—C11—O7	−3.9 (7)	C24—O28—C213—O29A	31 (3)
C10—O6—C11—C12	174.3 (4)	C24—O28—C213—C214	−176.2 (6)
C4—O8—C13—O9	−6.1 (7)	C33—O31—C32—C31	5.7 (13)
C4—O8—C13—C14	172.9 (4)	C34—C31—C32—O31	−4.8 (14)
C1—S1—S2—C21	−80.25 (19)	C32—O31—C33—C34	−4.8 (13)
C25—O21—C21—C22	−66.6 (4)	O31—C33—C34—C31	1.7 (12)
C25—O21—C21—S2	175.1 (3)	C32—C31—C34—C33	1.9 (12)
S1—S2—C21—O21	−85.4 (3)		
